# Electroacupuncture as an adjunctive therapy for drug-refractory primary open-angle glaucoma: a CARE-compliant case report

**DOI:** 10.3389/fmed.2026.1835330

**Published:** 2026-06-08

**Authors:** Yiqing Zhang, Qianyue Chen, Sibiao Lu, Hantong Hu, Qi Zhang, Gaofeng Liu, Zhong Di

**Affiliations:** 1 The Third Clinical Medical College, Zhejiang Chinese Medical University, Zhejiang, China; 2 Department of Acupuncture and Moxibustion, The Third Affiliated Hospital of Zhejiang Chinese Medical University, Zhejiang, China; 3 Department of Ophthalmology, The Third Affiliated Hospital of Zhejiang Chinese Medical University, Zhejiang, China

**Keywords:** case report, electroacupuncture, intraocular pressure, primary open-angle glaucoma, visual acuity

## Abstract

**Background:**

Primary open-angle glaucoma (POAG) is a progressive optic neuropathy; elevated intraocular pressure (IOP) is the only broadly modifiable risk factor. Some elderly patients remain uncontrolled on multi-drug regimens and are not immediate candidates for laser or filtering surgery. This CARE-compliant case report describes an integrative approach in such a patient.

**Case presentation:**

This case describes a 76-year-old woman with drug-refractory POAG and fluctuating IOP despite quadruple topical therapy. Adjunct electroacupuncture (EA, twice weekly) was initiated on March 28, 2025. IOP decreased from 12 mmHg in the right eye (OD) / 29 mmHg in the left eye (OS) to within target after the first two sessions and remained stable; best-corrected visual acuity (BCVA) improved from 0.4 (OD) / 0.25 (OS) to 0.5 in both eyes (OU). No major adverse events were observed. The observed clinical course was consistent with previously reported short-term IOP-lowering and ocular-perfusion effects of acupuncture in POAG.

**Conclusion:**

EA layered on top of standard care may be a feasible adjunct for short-term IOP stabilization and symptomatic improvement in drug-refractory POAG. However, given that topical antiglaucoma therapy was continued throughout the intervention and the inherent limitations of a single-case design, causal attribution cannot be established, and confirmation in randomized sham-controlled trials with longer follow-up is needed.

## 1 Introduction

Primary open-angle glaucoma (POAG) is a leading cause of irreversible blindness and presents with progressive retinal ganglion cell loss, optic-nerve head remodeling, and characteristic visual-field defects (1). IOP reduction is central to care, primarily achieved through escalating pharmacotherapy or surgical intervention. However, a subset of elderly patients may exhibit suboptimal responses to multiple medications due to tolerability or efficacy issues, and may also be poor candidates for or decline invasive procedures due to associated risks, creating a therapeutic gap.

Acupuncture, a long-standing modality of traditional Chinese medicine, has gained growing research interest in ophthalmology. Preliminary clinical studies, including randomized trials, suggest that acupuncture or electroacupuncture may transiently reduce intraocular pressure (2) and enhance ocular perfusion (3) in glaucoma patients, indicating potential adjunctive value. Although evidence remains limited and heterogeneous, these findings support exploring acupuncture as a complementary option for individuals with suboptimal responses or tolerability to standard glaucoma therapies. The present report follows the CARE (CAse REport) guidelines for transparent and structured reporting of this single case.

## 2 Case presentation

### 2.1 Patient information

A 76-year-old retired woman (height 158 cm, body weight 56 kg, body mass index 22.4 kg/m^2^) residing in an urban community of Hangzhou, China, was diagnosed with POAG in May 2022, with severe bilateral visual-field loss and optic-nerve atrophy. She had completed secondary education, and reported no occupational ocular trauma, no tobacco use, and no regular alcohol consumption. She had a history of recurrent iridocyclitis, which was active between 2013 and 2020. Other past procedures included bilateral cataract extraction with intraocular lens implantation in November 2019, followed by Nd:YAG posterior capsulotomy in September 2021. Autoimmune serology had once shown soluble nuclear antigen antibody positivity and weak anticentromere positivity. Her medical comorbidities included well-controlled essential hypertension; she had no known diabetes mellitus, coronary artery disease, cerebrovascular disease, chronic kidney disease, or coagulation disorder. She denied any history of allergy to medications, and had received no systemic corticosteroids, immunosuppressants, or anticoagulants within the preceding 12 months. There was no family history of glaucoma, and no first-degree relatives were known to have any other inherited ocular disease.

On March 28, 2025, the patient was referred to our hospital presenting with ocular fullness and blurred vision. Upon presentation, despite a stepwise escalation to quadruple topical therapy (tafluprost 0.0015% once nightly, carteolol 0.5% twice daily, brimonidine tartrate 0.2% twice daily, and brinzolamide 1% twice daily), she reported ocular fullness and blurred vision. Baseline IOP was 12 mmHg (OD) / 29 mmHg (OS); BCVA 0.4 (OD) / 0.25 (OS). Standard automated perimetry showed diffuse severe defects; OCT revealed marked thinning of the RNFL, showing an average peripapillary thickness of 61 μm (OD) and 68 μm (OS), alongside elevated cup-to-disc ratios of 0.94 (OD) and 0.85 (OS).

### 2.2 Diagnostic assessment

A comprehensive diagnostic work-up was performed prior to the initiation of electroacupuncture. Ocular examinations mainly included slit-lamp biomicroscopy, gonioscopy, intraocular pressure measurement, dilated fundus examination, optical coherence tomography of the retinal nerve fiber layer and optic-nerve head, and 24–2 standard automated perimetry. Laboratory investigations performed at presentation included complete blood count, fasting blood glucose, glycated hemoglobin, hepatic and renal panels, serum electrolytes, C-reactive protein, erythrocyte sedimentation rate, antinuclear antibody panel, and serological screening for syphilis, hepatitis B, hepatitis C, and human immunodeficiency virus; all results were within or close to reference ranges, with the previously documented soluble nuclear antigen antibody positivity and weak anticentromere positivity being the only persistently abnormal findings. Carotid Doppler ultrasonography revealed no haemodynamically significant stenosis.

Differential diagnoses mainly included angle-closure glaucoma, secondary uveitic glaucoma, and normal-tension glaucoma. Angle-closure glaucoma was excluded by an open anterior chamber angle on gonioscopy bilaterally. Active uveitic glaucoma was considered unlikely because slit-lamp examination showed no cells, flare, or keratic precipitates at presentation or throughout the follow-up period. Normal-tension glaucoma was excluded by the persistently elevated IOP in the left eye despite maximal tolerated medical therapy.

The patient was therefore given a final diagnosis of advanced, drug-refractory primary open-angle glaucoma (POAG) based on the combination of an open anterior chamber angle on gonioscopy, characteristic severe visual-field defects, corresponding retinal nerve fiber layer (RNFL) thinning on optical coherence tomography, and persistently elevated intraocular pressure (IOP) despite maximal tolerated medical therapy, in the absence of features supporting alternative diagnoses. Although no signs of active intraocular inflammation were observed during the treatment period, the patient’s history of recurrent uveitis suggested a chronic inflammatory and microcirculatory background, which could plausibly have contributed, although this cannot be proven in the present case, to trabecular dysfunction and suboptimal responsiveness to pharmacological therapy. This inflammatory milieu was therefore considered an aggravating factor rather than a primary diagnostic feature.

### 2.3 Therapeutic intervention

**Figure 1:**
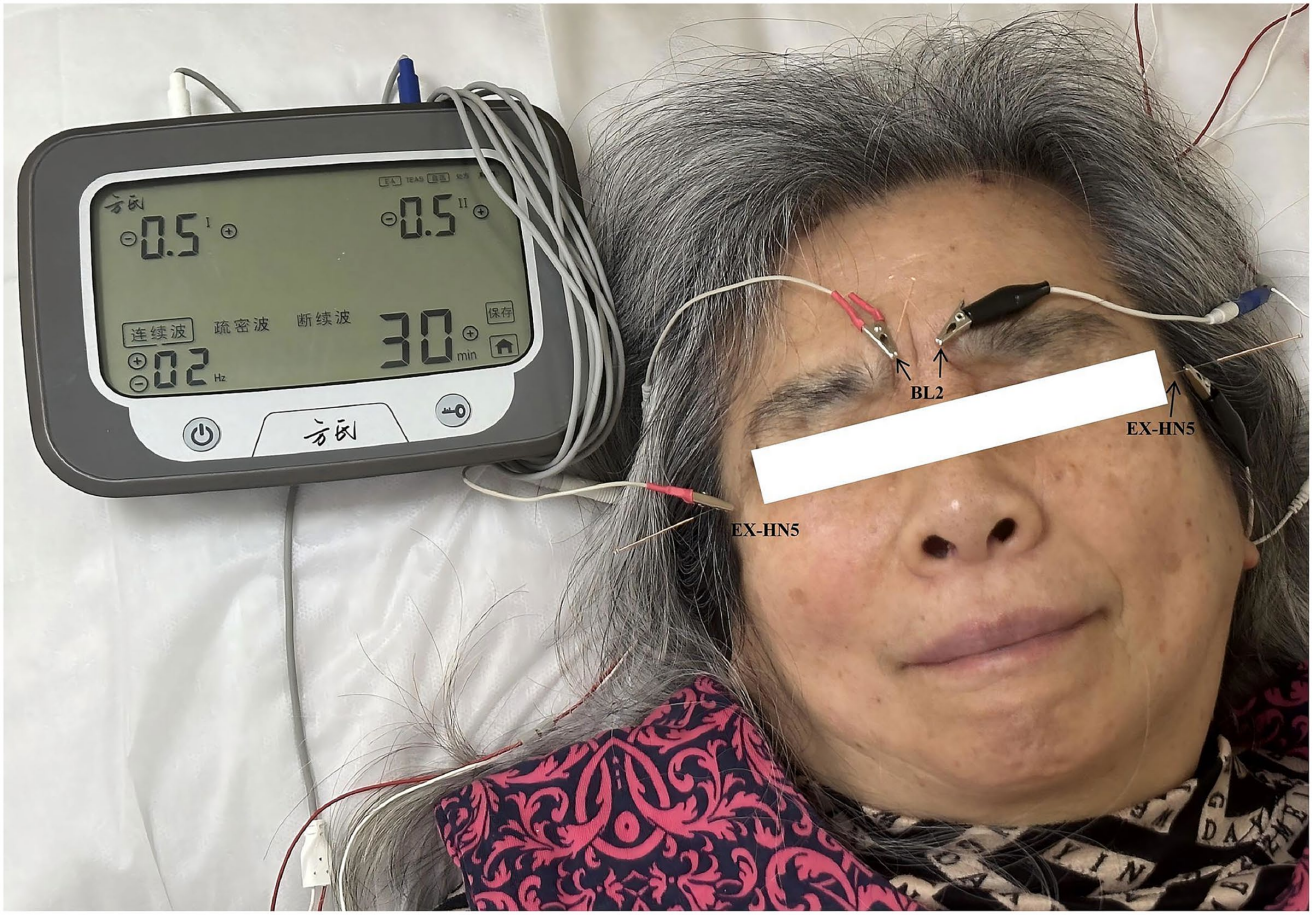
Electroacupuncture setup showing point connection (BL2 and EX-HN5) and instrument parameter settings (continuous wave, 2 Hz).

Treatment was administered in an outpatient integrative medicine clinic under standard aseptic conditions by a licensed acupuncturist with more than 10 years of clinical experience in ophthalmic electroacupuncture. After routine skin preparation with 75% medical ethanol swabs, single-use sterile filiform acupuncture needles made of austenitic 304 stainless steel with red-copper handles (Hwato, Suzhou Medical Appliance Factory, Suzhou, China) were used. Acupuncture points were localized according to the WHO Standard Acupuncture Point Locations in the Western Pacific Region (2008) (4): Cuanzhu (BL2) at the medial end of the eyebrow, Taiyang (EX-HN5) in the temporal fossa, approximately 1 cun posterior to the midpoint between the lateral canthus and eyebrow, and Fengchi (GB20) at the craniocervical junction inferior to the occipital bone, between the sternocleidomastoid and trapezius muscles. The upper-lateral line of the occiput (MS13)—a scalp acupuncture zone corresponding to the visual cortex, located 0.5 cun lateral and parallel to the Upper-Middle Line of the Occiput (GV18 to GV17)—was located according to the International Standard Scalp Acupuncture (ISSA) system (5, 6). For patient comfort, MS13 and GB20 were needled first with the patient seated, before transitioning to the supine position for BL2 and EX-HN5 needling with electroacupuncture application. Needle sizes were selected according to anatomical location and needling technique: 0.25 mm × 25 mm for BL2, EX-HN5, and MS13 (the latter inserted subcutaneously along the scalp plane), and 0.25 mm × 40 mm for GB20. The De-Qi sensation (a localized soreness, distension, or heaviness reported by the patient) was elicited at each point. Electroacupuncture was delivered using a FANGS-100 Percutaneous Acupoint Therapeutic Apparatus (Hangzhou Hercule Medical Equipment Co., Ltd., Hanzhou, China), with a continuous wave at 2 Hz and the intensity adjusted to patient tolerance (approximately 0.5–1.0 mA in this patient, titrated to elicit a visible but non-painful muscle twitch) (Figure 1). Ipsilateral electrical coupling was applied between BL2 and EX-HN5 on each side. MS13 and GB20 were needled manually without electrical stimulation. Needles were retained for 30 min per session, with treatments administered twice weekly for a total of 10 sessions.

The patient’s pre-existing quadruple topical antiglaucoma therapy was continued at the initiation of electroacupuncture. Importantly, no *a priori* protocol-driven tapering schedule was established before treatment initiation; rather, the decision to taper was made in a response-contingent manner, jointly by the ophthalmologist and the patient, based on observed clinical stability. Specifically, A stepwise medication tapering strategy was implemented contingent upon achieving and maintaining a target intraocular pressure (IOP) of ≤14 mmHg for approximately 10 days, following the principle of reducing one agent at a time with close clinical monitoring. The order of withdrawal (brinzolamide first, followed by carteolol) prioritized agents associated with the most prominent systemic and ocular surface side-effect burden in this elderly patient. The patient was explicitly counseled that, should IOP rebound to >14 mmHg at any monitoring visit, the withdrawn medication would be promptly reinstated. After completing the 10-session EA course, the patient was followed for an additional 3 months to monitor clinical outcomes.

### 2.4 Outcomes and follow-up

**Figure 2:**
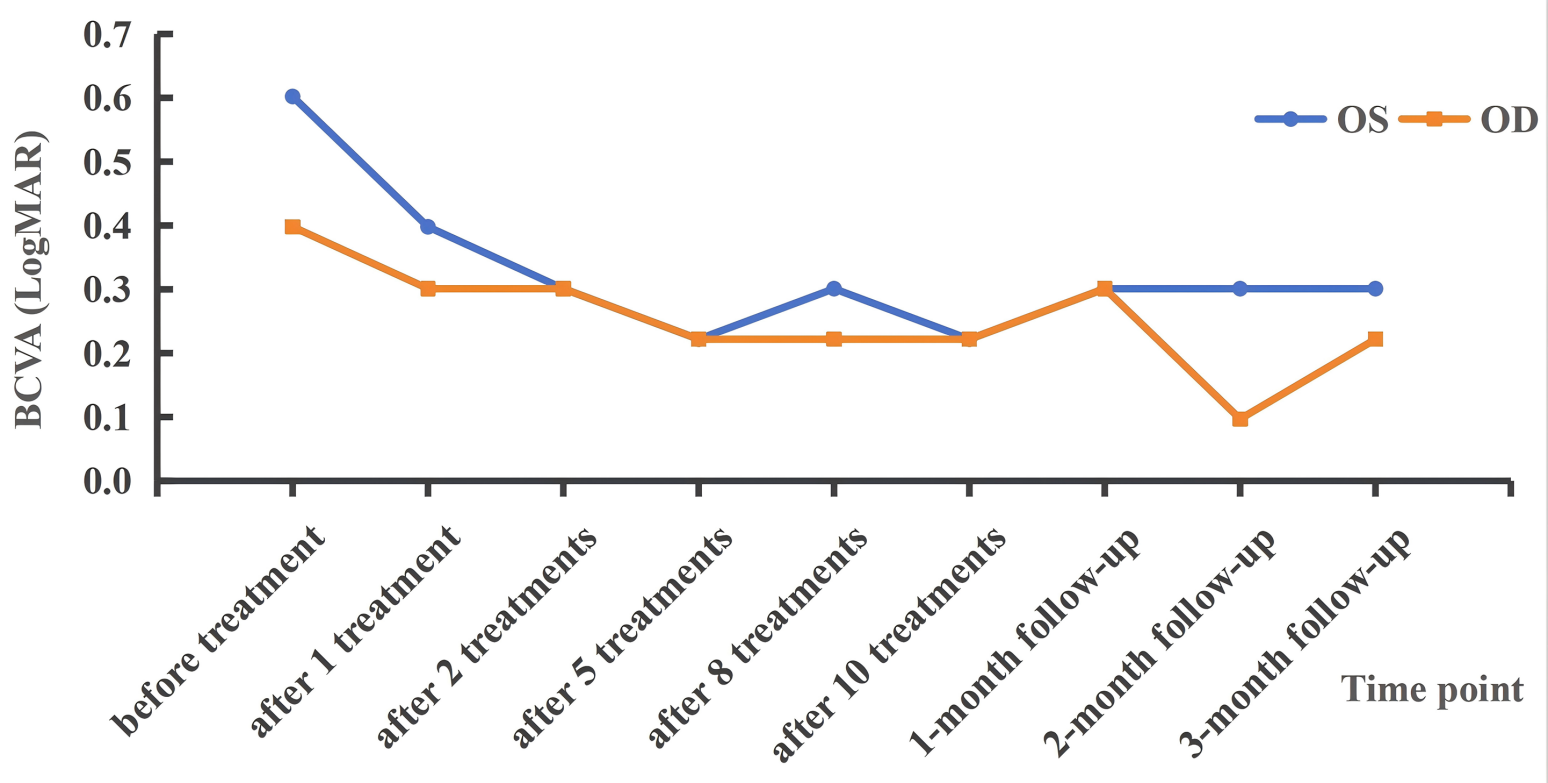
Best-corrected visual acuity (BCVA) changes during the study period, expressed in LogMAR units (lower values indicate better vision). The right eye (OD, orange squares) improved from 0.40 LogMAR (decimal 0.4) to 0.22 LogMAR (decimal 0.6) after 10 sessions, peaked at 0.10 LogMAR (decimal 0.8) at 2-month follow-up, and stabilized at 0.22 LogMAR (decimal 0.6) by 3 months. The left eye (OS, blue circles) improved from 0.60 LogMAR (decimal 0.25) at baseline to 0.22 LogMAR (decimal 0.6) after 10 sessions and remained stable at approximately 0.30 LogMAR (decimal 0.5) throughout the follow-up. This corresponds to a gain of ~3–4 lines on the visual acuity chart.

Intraocular pressure (IOP), best-corrected visual acuity (BCVA), and visual fields were selected as core outcomes, reflecting modifiable risk control, functional status, and disease progression, respectively, consistent with glaucoma monitoring guidelines. IOP was measured immediately after each treatment session and monthly during follow-up using a calibrated non-contact tonometer (Nidek NT-530P), with two consecutive seated readings averaged per eye. In the present case, non-contact tonometry was chosen on pragmatic grounds (rapid, non-invasive, and well tolerated for the multiple per-session and monthly measurements required by the study schedule), with the same device and operator used throughout to minimize systematic bias. BCVA was assessed at the same time points using a Snellen chart, with 2–3 repeated measurements recorded in decimal notation (with LogMAR equivalents used in Figure 2 for between-eye comparison). Visual fields were evaluated at the 3-month follow-up using a Humphrey perimeter under standardized conditions.

**Figure 3:**
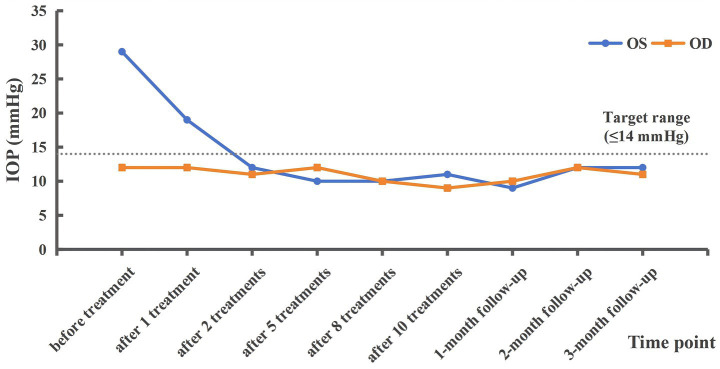
Intraocular pressure (IOP) changes during electroacupuncture treatment and follow-up. The right eye (OD, orange squares) maintained IOP within the target range (≤14 mmHg, dashed line) at all time points (range: 9–12 mmHg). The left eye (OS, blue circles) decreased from 29 mmHg at baseline to ≤14 mmHg after 2 sessions, remaining stable at 9–12 mmHg throughout follow-up.

At baseline, IOP was 12 mmHg in the right eye (OD) and 29 mmHg in the left eye (OS). IOP declined to the target range (≤14 mmHg) after the first two EA sessions. Upon confirming sustained stability at this target over the following 10 days, a stepwise drug taper was initiated at the 5th session with the discontinuation of brinzolamide. After another 10-day monitoring period confirmed maintained control, carteolol was withdrawn at the 8th session. Consequently, the patient was stabilized on dual therapy (tafluprost and brimonidine) from the 8th session onward. IOP remained within the target range throughout the remainder of the 10-session course and during the 3-month follow-up period (Figure 3). BCVA improved from 0.4 (OD) / 0.25 (OS) at baseline to ≥ 0.5 (OU) by mid-course, and this improvement was sustained thereafter (Figure 2, expressed in LogMAR units; lower values indicate better vision). Standard automated perimetry performed at the 3-month follow-up visit showed that the pre-existing severe diffuse visual-field defects remained stable without obvious progression. In parallel, the patient completed all 10 scheduled sessions, tolerated the treatment well without any adverse events, and reported relief of ocular fullness, improved visual clarity, and a positive treatment experience throughout the intervention and follow-up. A detailed timeline of treatment events and outcomes is provided in Table 1.

**Table 1:** Timeline of treatment, interventions, and clinical outcomes.

Time point	Key event	IOP (mmHg) OD/OS	BCVA (LogMAR) OD/OS	Medications
Baseline (Mar 28, 2025)	Initial presentation; quadruple therapy ongoing	12/29	0.40/0.60	Tafluprost, carteolol, brimonidine, brinzolamide
After 1 EA sessions (Apr 1, 2025)	IOP began to decrease	12/19	0.30/0.40	Tafluprost, carteolol, brimonidine, brinzolamide
After 2 EA sessions (Apr 4, 2025)	IOP first reached target range (≤14 mmHg)	11/12	0.30/0.30	Tafluprost, carteolol, brimonidine, brinzolamide
After 5 EA sessions (Apr 18, 2025)	Brinzolamide discontinued (stepwise taper initiated)	12/10	0.22/0.22	Tafluprost, carteolol, brimonidine
After 8 EA sessions (Apr 25, 2025)	Carteolol withdrawn; stabilized on dual therapy	10/10	0.22/0.30	Tafluprost, brimonidine
After 10 EA sessions (May 2, 2025)	EA course completed	9/11	0.22/0.22	Tafluprost, brimonidine
1-month follow-up (Jun 2, 2025)	Sustained IOP and BCVA	10/9	0.30/0.30	Tafluprost, brimonidine
2-month follow-up (Jul 3, 2025)	Peak BCVA in right eye	12/12	0.10/0.30	Tafluprost, brimonidine
3-month follow-up (Aug 3, 2025)	Final follow-up; stable outcomes	11/12	0.22/0.30	Tafluprost, brimonidine

Safety monitoring was performed at every treatment session and at each follow-up visit. At each session, the acupuncturist inspected the periocular skin, eyelids, and conjunctiva for haematoma, bleeding, or signs of infection; the patient was asked to report any pain, light-headedness, syncope, palpitations, nausea, or transient visual disturbance both during and immediately after needle retention. A standardized adverse-event checklist (covering needling-related events such as fainting, broken needle, severe pain, haematoma, infection, and ocular events such as conjunctival hemorrhage, eyelid swelling, or transient visual blurring) was completed by the acupuncturist after each session. Slit-lamp biomicroscopy was repeated at the end of the 10-session course and at each monthly follow-up visit. No needling-related, ocular, or systemic adverse events were recorded throughout the intervention or the 3-month follow-up.

### 2.5 Patient perspective

The patient was invited to share her perspective in a semi-structured interview at the end of the 3-month follow-up. Her account, summarized here with her written permission, is reproduced in her own framing: “Before starting the acupuncture treatment, I felt a heavy, pressing fullness around my left eye, and my vision was so blurred that reading became difficult. I was anxious that I would have to undergo surgery despite my age. After a few sessions, the heaviness eased and I could see the television and read large print more clearly. I felt reassured when my ophthalmologist agreed to reduce one of my eye drops, because I had been worried about the side effects of using so many medications. The needling itself was tolerable—only a mild soreness—and I never felt unwell during or after the sessions. Overall, I felt my eye condition was being managed in a more comprehensive way, and I would be willing to consider this combined approach again if needed.”

Notably, the patient’s account is included to provide transparency regarding her subjective experience and to support CARE compliance; it should be interpreted as a personal narrative rather than as objective evidence of treatment efficacy.

## 3 Discussion

This case describes a 76-year-old woman with advanced, drug-refractory primary open-angle glaucoma and a concomitant long-standing history of recurrent uveitis (with documented activity between 2013 and 2020). After 10 sessions of electroacupuncture (EA), her intraocular pressure (IOP) fell into the target range and remained stable, permitting stepwise tapering of topical therapy without IOP rebound—a finding that hypothesis-generating and suggests EA may be a useful adjunct in selected refractory cases, although causal attribution cannot be established from a single uncontrolled observation. Best-corrected visual acuity (BCVA) improved to and was maintained at ≥0.5, with no serious adverse events. Visual field defects did not show definitive reversal. Importantly, topical antiglaucoma therapy was continued throughout the EA intervention and was tapered only in a response-contingent manner; the observed IOP reduction must therefore be interpreted in the context of ongoing pharmacological treatment, and the relative contribution of EA versus continued (although progressively reduced) topical therapy cannot be disentangled in this case.

The pathophysiology of POAG involves progressive retinal ganglion cell loss and optic nerve head remodeling, with IOP as the primary modifiable risk driver (7). Therefore, the cornerstone of clinical management is to reduce and maintain IOP within a personalized target range —typically aiming for a 20–30% reduction from baseline initially, with a lower target (e.g., 30–40% or more) considered for advanced or rapidly progressing cases (8). However, in patients with specific backgrounds such as potential chronic inflammation—illustrated by our case with a history of uveitis— trabecular dysfunction and ocular perfusion instability have been hypothesized to interact, contributing to a proposed but not yet causally proven “IOP-inflammation-ischemia” vicious cycle. Some experimental and clinical evidence is consistent with the view that neuroinflammation, glial activation, and impaired vascular autoregulation are associated with increased vulnerability of the optic nerve head to pressure-related stress, thereby facilitating ongoing neurodegeneration (9, 10). Consequently, managing these refractory cases is challenging as long-term combination therapy is prone to aggravating ocular (11) and systemic side effects (e.g., *β*-blocker-associated cardiopulmonary events (12)), undermining adherence and limiting further pharmacotherapeutic options. While laser and filtering/drainage surgeries are effective in lowering IOP, they carry risks such as transient IOP spikes, anterior chamber inflammation, hypotony, and bleb-related infections, complicating shared decision-making (13, 14). Precisely due to these limitations of conventional treatments, there is a clinical need to explore safe and effective adjuvant therapies for such patients.

Acupuncture, including modalities like electroacupuncture and scalp acupuncture, has been proposed as a possible adjuvant intervention in glaucoma management. Small-sample randomized controlled trials have reported that acupuncture or EA can induce short-term IOP reduction at specific time points (2), with some studies observing improvements in ocular perfusion parameters. However, the magnitude and durability of these effects vary substantially across studies note that the overall evidence remains limited (3), necessitating confirmation through larger, more rigorous trials. Regarding potential mechanisms, EA with specific parameters (e.g., 2 Hz continuous wave) has been reported in multiple animal and human disease model studies to upregulate neurotrophic factors such as BDNF and mitigate inflammatory responses following ischemia–reperfusion, providing a biological hypothesis, although not yet a confirmed mechanism, for its neuroprotective utility (15, 16). Scalp acupuncture, based on the “cortical localization-scalp correspondence” theory, is supported by functional imaging studies and reviews indicating its potential to improve local cerebral blood flow and functional connectivity in related cortical areas (17, 18). Furthermore, from an anatomical perspective, the EX-HN5 (Taiyang) region features an anastomotic network between the frontal branch of the superficial temporal artery and branches of the ophthalmic artery (supraorbital/supratrochlear), providing a structural basis for influencing periocular circulation and local autonomic reflex pathways to affect the eye (19, 20). These multi-pathway lines of evidence, although largely indirect, are compatible with the hypothesis that acupuncture may offer a complementary strategy for the comprehensive management of complex glaucoma by targeting IOP reduction, perfusion improvement, and possibly neuroprotection; these mechanistic considerations should be regarded as exploratory rather than established.

Based on the above mechanistic insights, we formulated a composite electroacupuncture (EA) protocol for this patient, who continued her standard IOP-lowering medication. For local periocular regulation, BL2 and EX-HN5 were chosen as paired points for electroacupuncture based on their direct vascular association with the eye. BL2 adjoins the supraorbital neurovascular bundle, while EX-HN5 overlies the anastomosis between superficial temporal and ophthalmic arteries. As a key acupoint for ocular disorders, EX-HN5 and its combinations have been associated with improved ocular perfusion. Takayama et al. further confirmed that a compound acupuncture regimen including BL2 and EX-HN5 significantly reduced IOP and ameliorated retrobulbar circulation in patients with OAG (21). Although the individual efficacy of each point cannot be isolated, their combined use may benefit ocular hemodynamic regulation and IOP control. Additionally, GB20 at the craniocervical junction (22) was selected, as its stimulation modulates posterior circulation—as evidenced by altered vertebral-basilar artery blood flow velocity (23, 24)—and may thereby indirectly optimize the blood supply to the optic nerve. Informed by scalp acupuncture research (25), bilateral stimulation of MS13 was applied to enhance cerebral blood flow and functional connectivity in the corresponding parieto-occipital cortical areas. Based on evidence that low-frequency EA promotes neural plasticity (26) and on the short-term IOP observation window reported in prior randomized studies (2), the EA parameters were set as follows: 2 Hz continuous wave with intensity adjusted to patient tolerance, 30 min per session, administered twice weekly for a total of 10 sessions. This integrated protocol was designed to address, in a complementary manner, central regulation, local ocular circulation, and putative neuroprotective pathways relevant to the complex pathological mechanisms of this refractory glaucoma case.

The rapid decline and sustained maintenance of IOP in this case provide a preliminary signal of clinical feasibility for this adjuvant intervention in achieving short-term IOP stabilization and symptom relief. However, as an uncontrolled single-case report, this study is particularly susceptible to influences such as observer bias, placebo effects, spontaneous fluctuation of IOP, and measurement variability inherent to non-contact tonometry; therefore, causal interpretation must be made with caution. Furthermore, because topical antiglaucoma therapy was continued throughout the intervention, the observed IOP reduction cannot be attributed solely to electroacupuncture. Future validation should employ randomized, sham-controlled designs with blinded assessment and standardized IOP measurement using Goldmann applanation tonometry. This study has several additional limitations beyond those inherent to a single-case design. First, IOP was not systematically sampled at multi-timepoints (e.g., immediate/≤5 min, 30–120 min, 120 min), hindering the characterization of EA’s effect on transient and peak IOP. Future studies should implement Goldmann applanation tonometry as the reference standard with continuous/multi-timepoint monitoring. Second, follow-up OCT scans and quantitative retinal perfusion metrics (e.g., OCT-Angiography vessel density or orbital Doppler) were not obtained, limiting interpretation of structure-perfusion-function coupling and potential neuroprotective effects. Subsequent research should incorporate above objective measures to verify any putative microcirculatory benefits beyond IOP modulation (8). Third, the 3-month follow-up is sufficient to demonstrate short-term feasibility and tolerability but is too brief to evaluate slowing of visual-field progression, structural neuroprotection, or the durability of IOP control after medication tapering; longer-term observation (ideally ≥12 months) is required to clarify any sustained effects. Fourth, as a single-patient observational report, the findings cannot be generalized to the broader POAG population and should be regarded as hypothesis-generating only.

In summary, this case suggests that for elderly patients with refractory POAG and suboptimal response to pharmacotherapy who are at higher surgical risk, adjunctive EA under close monitoring may offer possible adjunctive value in short-term IOP stabilization and symptom improvement. This finding indicates that within the conventional treatment framework, EA, as a potentially safe and accessible adjuvant strategy, warrants further investigation. The present report does not establish efficacy, and the conclusions—particularly regarding definitive structural/functional benefits and longer-term safety—must be verified in large, rigorous, sham-controlled randomized trials with standardized IOP measurement and adequate follow-up duration.

## Data Availability

The original contributions presented in the study are included in the article/supplementary material, further inquiries can be directed to the corresponding authors.
